# Angiomyolipoma of the kidney—Clinicopathological analysis of 52 cases

**DOI:** 10.3389/pore.2022.1610831

**Published:** 2023-01-09

**Authors:** Zsuzsanna Fejes, Fanni Sánta, Alex Jenei, István Előd Király, Linda Varga, Levente Kuthi

**Affiliations:** ^1^ Department of Radiology, Albert Szent-Györgyi Medical School, University of Szeged, Szeged, Hungary; ^2^ Department of Pathology, Albert Szent-Györgyi Medical School, University of Szeged, Szeged, Hungary; ^3^ Department of Pathology and Experimental Cancer Research, Semmelweis University, Budapest, Hungary; ^4^ Department of Urology, Albert Szent-Györgyi Medical School, University of Szeged, Szeged, Hungary; ^5^ Department of Oncotherapy, Albert Szent-Györgyi Medical School, University of Szeged, Szeged, Hungary

**Keywords:** AML, angiomyolipoma, kidney tumor, tuberous sclerosis, nephrectomy

## Abstract

The renal angiomyolipoma (AML) is a benign tumor characteristically composed of fat, smooth muscle tissue, and vessels. We collected AMLs from our nephrectomy database, reclassified them according to their histological appearance, recorded the demographic, clinical, and pathological parameters, and compared them with oncocytoma (RO) and renal cell carcinoma (RCC). Immunohistochemistry was ordered in 41 cases. In 2224 nephrectomies, we found 52 AMLs with a 53 mm median size. The mean age was 52.76. Forty-eight tumors were sporadic, while four were hereditary. The revision resulted in 31 classic, 13 leiomyoma-like, five lipoma-like, two epithelioid, and one AML with epithelial cysts. SMA was diffusely positive, except for the epithelioid type, while MelanA harbored stronger expression than HMB45. AML was more frequent in females and appeared ten and 7 years earlier than RO and RCC, respectively. The follow-up time was 7.42 years, and neither tumor-related death nor relapse occurred. AML is rare in nephrectomies and develops primarily in females in their 50s with an average size of 50–60 mm at the surgery. The histological appearance in order of frequency is classic, leiomyoma-like, lipoma-like, epithelioid, and cystic. The MelanA, HMB45, and SMA immunohistochemistry can support the light-microscopic findings.

## Introduction

The angiomyolipoma (AML) is a benign tumor occurring mainly in the kidney and belongs to the perivascular epithelioid cell tumors (PEComa) [1, 2]. Generally, AML is composed of thick-walled vessels, adipose, and smooth muscle tissue in various distributions [3], but occasionally, the tumor has cystic, leiomyoma-like, lipoma-like, or epithelioid appearance [4]. In contrast to classic AML, the latter has malignant potential, and a certain number of these cases may relapse or give distant metastasis [4]. AML can be sporadic or hereditary and linked to tuberous sclerosis (TSC) [5]. Sporadic tumors are four times more frequent in females, related to hormonal causes [6, 7]. Hereditary cases develop in younger individuals and have no gender predilection [4, 8]. Genetically speaking, AMLs are characterized by the biallelic inactivation of *TSC1* or *TSC2* genes [5, 9], which encode the hamartin and tuberin, respectively [5, 10]. These proteins, along with TBC1D7, build up the TSC1-TSC2 complex that regulates the cellular metabolism, protein synthesis, and cell cycle *via* the mTOR pathway [11]. AML occurs approximately in 1% of the nephrectomy specimens [4]. Most of the sporadic cases are discovered accidentally, but infrequently (2.2%), they may cause hemorrhage, which positively correlates with the tumor size [12, 13]. In a typical clinical scenario, the imaging technics can differentiate AMLs from renal cell tumors [14, 15], and for these cases, the radiologic follow-up might be adequate and beneficial. However, the usual sensitivity of radiological techniques is inappropriate in epithelioid and leiomyoma-like morphology [16]. Surgical removal is needed for tumor-related symptoms, compression of the adjacent structures, and hemorrhagic complications [17]. If applicable, nephron-sparing techniques should be used [17]. For classic AML, the pathological diagnosis is usually straightforward [4], but tumors with epithelioid [18] and leiomyoma-like [4] morphology often require extensive immunohistochemical examinations to exclude renal cell carcinoma (RCC), metastasis, and sarcoma. Multifocality and bilaterality are worrisome features for TSC; consequently, in the cases, clinical genetic consultation along with the investigation of the *TSC1* and *TSC2* are necessary [19]. Peripheral blood and buccal smear can be the source for the germline testing of the genes mentioned above [19]. We collected 52 consecutively removed AMLs and analyzed the patients’ clinical characteristics and the tumors’ pathological features.

## Materials and methods

### Case selection and pathological revision

AML cases were collected from the archive of the Department of Pathology, Albert Szent-Györgyi Medical School, University of Szeged. Two pathologists (AJ and LK) reviewed all hematoxylin eosin-stained slides and available immunohistochemical staining. They reclassified the cases according to the current classification scheme, and the subtypes were as follows: classic AML, AML with epithelial cysts (AMLEC), lipoma-like AML, leiomyoma-like AML, oncocytic AML, and epithelioid AML (eAML) [4]. In this study, solely nephrectomy samples were enrolled; therefore, biopsy and autopsy cases were excluded. The demographic data (age and gender) along with the main clinical features (symptoms and syndromic background) were collected. The tumors’ size, laterality, and multifocality were registered based on the original pathology report. These characteristics were compared with renal cell carcinoma (RCC) and oncocytoma (RO).

### Immunohistochemistry

All immunohistochemical stains available in 22 tumors were reviewed. By using tissue microarray technique, another 19 AMLs were stained by MelanA (Labvision, clone A103, mouse monoclonal antibody, dilution 1:200), HMB45 (Cell Marque, clone hmb-45, mouse monoclonal antibody, dilution: 1:200), and SMA (Cell Marque, clone 1a4, mouse monoclonal antibody, dilution: 1:300). Two 2-mm-thick tissue cores represented the tumors. The reactions were evaluated in a semiquantitative fashion (0% positivity = negative; 1%–50% positivity = +; 51%–100% positivity = ++). The FFPE blocks were unavailable in eleven cases; hence, no immunohistochemistry was performed for these tumors.

### Statistical analysis

For parametric and non-parametric tests, the SPSS software package was applied, and the differences were deemed significant if *p* < .05.

## Results

### Clinical aspects

**FIGURE 1:**
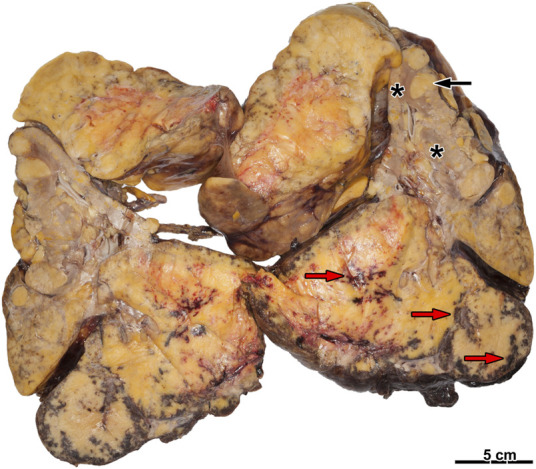
Ruptured angiomyolipoma with hemorrhage. There is a fatty tumor on the cut surface with several foci of hemorrhage (red arrow). On the other hand, several smaller tumor nodules are present (black arrow). The renal parenchyma is hard to recognize (asterisks).

Fifty-two AML cases were diagnosed from 2224 nephrectomy specimens. The mean age of all patients was 52.76 years (range 27–76 years). In males, 7 tumors, while, in females, 45 AMLs occurred (female-to-male ratio: 6.42:1). Forty-eight tumors were sporadic in our data set, and four were linked to TSC. The mean ages of sporadic and TSC cases were 54.04 and 37.75, respectively. One tumor developed in polycystic kidney disease, and another one evolved in a graft kidney. Tumor rupture and hemorrhagic complications occurred in eight patients (shown in Figure 1), and in one case, a hemorrhagic shock was developed as well. Fourteen tumors were resected, while total and radical nephrectomy was carried out in 30 and 8 patients, respectively. The median follow-up time was 2.64 years, and neither local relapse nor tumor-related death was registered. The patients’ clinical characteristics are summarized in Table 1.

**TABLE 1:** The clinical features of the cases investigated.

Case	Age	Sex	Signs/Circumstances of discovery	Tuberous sclerosis	Surgery	Additional data
1	52	F	No data	No	Radical nephrectomy	-
2	66	F	Incidental finding at cholecystectomy	No	Tumor resection	-
3	73	F	Tumor rupture	No	Total nephrectomy	-
4	36	F	No data	No	Total nephrectomy	-
5	46	F	Tumor rupture	No	Total nephrectomy	-
6	55	F	Tumor rupture	No	Total nephrectomy	-
7	58	F	Retroperitoneal hemorrhage	No	Total nephrectomy	-
8	33	F	No data	Yes	Total nephrectomy	-
9	26	F	No data	No	Total nephrectomy	-
10	49	F	No data	No	Tumor resection	-
11	26	F	Surgical finding at kidney transplantation	No	Total nephrectomy	-
12	45	F	Incidental finding at SLE examination	No	Tumor resection	-
13	34	F	No data	Yes	Radical nephrectomy	-
14	46	F	No data	No	Total nephrectomy	-
15	53	F	No data	No	Radical nephrectomy	-
16	47	F	No data	No	Tumor resection	-
17	62	F	No data	No	Total nephrectomy	-
18	60	F	No data	No	Total nephrectomy	-
19	52	F	Tumor rupture	No	Radical nephrectomy	-
20	49	F	Incidental finding on abdominal US	No	Total nephrectomy	-
21	49	F	At the examination of hemorrhoids	No	Radical nephrectomy	-
22	60	F	Tumor rupture	No	Total nephrectomy	-
23	57	F	No data	No	Radical nephrectomy	-
24	60	F	Hematuria	No	Total nephrectomy	-
25	67	F	No data	No	Total nephrectomy	-
26	35	F	No data	No	Total nephrectomy	-
27	56	M	At the examination of kidney stones	No	Tumor resection	-
28	31	F	At the examination of PCOS	No	Tumor resection	-
29*	40	F	Hemorrhagic shock	Yes	Total nephrectomy	-
30	58	F	Tumor rupture	No	Total nephrectomy	Evolved in horseshoe kidney
31	43	M	At the examination for kidney transplantation	No	Radical nephrectomy	Evolved in polycystic kidney
32	66	F	No data	No	Total nephrectomy	-
33	73	M	At the examination of BPH	No	Tumor resection	-
34	76	F	Incidental finding on abdominal US	No	Tumor resection	-
35	54	M	At graft kidney’s follow-up	No	Tumor resection	Evolved in graft kidney
36	65	F	No data	No	Total nephrectomy	-
37	68	M	No data	No	Total nephrectomy	-
38	68	F	No data	No	Total nephrectomy	-
39	46	M	At the examination of urethral discharge	No	Total nephrectomy	-
40	75	F	No data	No	Total nephrectomy	Ipsilateral RO is present
41	69	F	No data	No	Tumor resection	-
42	56	F	No data	No	Tumor resection	-
43	74	F	No data	No	Total nephrectomy	Ipsilateral ccRCC is present
44*	43	F	Renal pain, tumor rupture	Yes	Radical nephrectomy	-
45	65	F	No data	No	Tumor resection	-
46	33	F	No data	No	Tumor resection	-
47	31	M	No data	No	Total nephrectomy	-
48	57	F	No data	No	Total nephrectomy	-
49	46	F	Renal colic	No	Total nephrectomy	Ipsilateral HOCT and ccRCC are present
50	60	F	At gastrointestinal examination	No	Tumor resection	-
51	54	F	Subcostal pain	No	Total nephrectomy	-
52	41	F	Incidental finding on abdominal US	No	Tumor resection	-

F, Female; M, Male; SLE, Systemic lupus erythematosus; US, Ultrasound; PCOS, Polycystic ovary syndrome; BPH, Benign prostatic hyperplasia; RO, Renal oncocytoma; ccRCC, Clear cell renal cell carcinoma; RCC-U, Renal cell carcinoma unclassified. * Case #29 and #44 are from the same patient.

### Pathological aspects

The median size of all tumors was 53 mm (range: 4–300 mm), but in cases with tuberous sclerosis, it was found to be 260 mm. The revision of the cases resulted in thirty-one classic, thirteen leiomyoma-like, five lipoma-like, two epithelioid, and one AMLEC. No oncocytic AML was seen. Synchronous tumors were observed in four cases (Table 1). Seven AMLs were multifocal. A renal sinus invasion was present in case #37 and #51 along with the infiltration of the renal vein in the latter. Both cases harbored epithelioid morphology. Besides, microscopic tumor necrosis was solely present in eAMLs. At least one melanocytic and smooth muscle marker was expressed in every case. Diffuse SMA-positivity was seen in all tumors except the eAMLs. Among the melanocytic markers, the MelanA was stronger (+ = 16; ++ = 18) compared to HMB45 (+ = 27, ++ = 7). Ki67 immunohistochemistry was available for six tumors, and the mean proliferation activity was about 1%. The morphological aspects are summarized in Table 2.

**TABLE 2:** The pathological characteristics of the cases investigated.

Case	Laterality	Focality	Size (mm)	Histological subtype	Immunohistochemistry
1	Right	Unifocal	20	Leiomyoma-like	MelanA: ++, HMB45: +, SMA: ++
2	Right	Unifocal	6	Leiomyoma-like	MelanA: ++, HMB45: +, SMA: ++
3	Right	Unifocal	No data	Classic	MelanA: -, HMB45: +, SMA: ++
4	Left	Unifocal	40	Classic	MelanA: +, HMB45: +, SMA: ++
5	Left	Unifocal	75	Classic	MelanA: ++, HMB45: +, SMA: ++
6	Right	Unifocal	20	Classic	MelanA: ++, HMB45: −, SMA: ++
7	Right	Unifocal	39	Leiomyoma-like	MelanA: ++, HMB45: +, SMA: ++
8	Left	Multifocal	No data	Classic	MelanA: ++, HMB45: ++, SMA: ++
9	Right	Unifocal	36	Leiomyoma-like	MelanA: ++, HMB45: ++, SMA: ++
10	Left	Unifocal	70	Lipoma-like	MelanA: +, HMB45: +, SMA: ++
11	Left	Multifocal	No data	Classic	Not performed
12	Right	Unifocal	22	Classic	MelanA: +, HMB45: +, SMA: ++
13	Left	Multifocal	200	Classic	MelanA: ++, HMB45: ++, SMA: ++
14	Left	Multifocal	25	Classic	Not performed
15	Right	Unifocal	90	Classic	Not performed
16	Right	Unifocal	20	Classic	Not performed
17	Left	Unifocal	17	Leiomyoma-like	HMB45: +, SMA: ++
18	Left	Unifocal	25	Classic	Not performed
19	Left	Unifocal	46	Classic	MelanA: ++, HMB45: ++, SMA: ++
20	Right	Unifocal	170	Lipoma-like	Not performed
21	Left	Unifocal	65	Classic	Not performed
22	Left	Unifocal	245	Classic	Not performed
23	Left	Unifocal	100	Classic	MelanA: +, HMB45: +, SMA: ++
24	Right	Unifocal	30	Leiomyoma-like	Not performed
25	Right	Unifocal	40	Classic	MelanA: +, HMB45: −, SMA: ++
26	Left	Unifocal	80	Classic	MelanA: −, HMB45: +, SMA: ++
27	Left	Unifocal	37	Leiomyoma-like	HMB45: +, SMA: ++
28	Right	Unifocal	40	Classic	MelanA: ++, HMB45: ++, SMA: ++, CD1a: −, Ki67: 1%
29	Right	Multifocal	300	Classic	MelanA: +, HMB45: +, SMA: ++
30	Right	Unifocal	155	Classic	MelanA: +, HMB45: +, SMA: ++
31	Left	Multifocal	6	Classic	MelanA: ++, HMB45: +, SMA: ++
32	Left	Unifocal	42	Leiomyoma-like	MelanA: +, HMB45: +, SMA: ++, h-Caldesmon: ++
33	Left	Unifocal	20	Classic	MelanA: +, HMB45: +, SMA: ++, CathepsinK: +
34	Left	Unifocal	31	Leiomyoma-like	MelanA: ++, HMB45: ++, SMA: ++ CD1a: −
35	Right	Unifocal	45	Lipoma-like	MelanA: ++, HMB45: ++, SMA: ++
36	Right	Unifocal	No data	Classic	Not performed
37	Left	Unifocal	40	Epitheloid	MelanA: +, HMB45: +, SMA: +, EMA: −, PAX2: −, S100: −, CD56: −, Ki67: 1%
38	Left	Unifocal	69	Classic	MelanA: ++, HMB45: -, SMA: ++
39	Right	Unifocal	20	Cystic	Epithel: CK7: ++, PAX8: ++, MNF116: ++, Stroma: MelanA: +, HMB45: +, SMA: ++, ER: +, PR: +, TLE1: −, S100: −, CD34: −, Ki67: <1%
40	Right	Unifocal	4	Classic	MelanA: +, HMB45: +, SMA: ++
41	Left	Unifocal	23	Leiomyoma-like	MelanA: +, HMB45: −, SMA: ++
42	Left	Unifocal	46	Classic	MelanA: ++, HMB45: −, SOX10: -, SMA: ++
43	Right	Unifocal	11	Leiomyoma-like	MelanA: +, HMB45: −, SMA: ++
44	Left	Unifocal	260	Classic	MelanA: ++, HMB45: +, SMA: ++
45	Right	Unifocal	30	Lipoma-like	Not performed
46	Graft	Unifocal	5	Lipoma-like	MelanA: +, HMB45: +, SMA: ++, S100: ++, CD34: -, Ki67: <1%
47	Left	Unifocal	52	Leiomyoma-like	HMB45: +, SMA: ++, CD117: +, Ki67: 1%–2%
48	Right	Unifocal	28	Classic	HMB45: +, SMA: ++
49	Left	Multifocal	4	Classic	MelanA: −, HMB45: ++, PAX8: −, SDHB: ++, β-Catenin: ++ (membrane), SMA: ++
50	Left	Unifocal	15	Leiomyoma-like	MelanA: ++, HMB45: +, SMA: ++, Ki67: 1%–2%
					MelanA: +, HMB45: -, SMA: +, CD68: +, CathepsinK: +, CD117: +, PAX2: −
51	Left	Unifocal	42	Epitheloid	PAX8: −, FH: ++, SDHB: ++, CA9: −, CK7: −, CD10: −, TFE3: −, GATA3: −
					PBRM1: ++, BAP1: ++
52	Left	Unifocal	55	Classic	MelanA: ++, HMB45: +, SMA: ++

− = negative (0% positivity), + = 1%–50% positivity, ++ = 51%–100% positivity.

HMB45, Human melanoma black 45; SMA, Smooth muscle actin; CD, Cluster of differentiation; EMA, Epithelial membrane antigen; PAX, Paired-box; ER, Estrogen receptor; PR, Progesterone receptor; TLE1, Transducin-like enhancer of split 1; SOX10, SRY-related HMG-box 10; SDHB, Succinate dehydrogenase B subunit; FH, Fumarate hydratase; CA9, Carbonic anhydrase 9; CK7, Cytokeratin 7; TFE3, Transcription factor 3E; GATA3, GATA-binding factor 3; PBRM1, Polybromo 1; BAP1, BRCA1 associated protein 1.

### Classic AML

**FIGURE 2:**
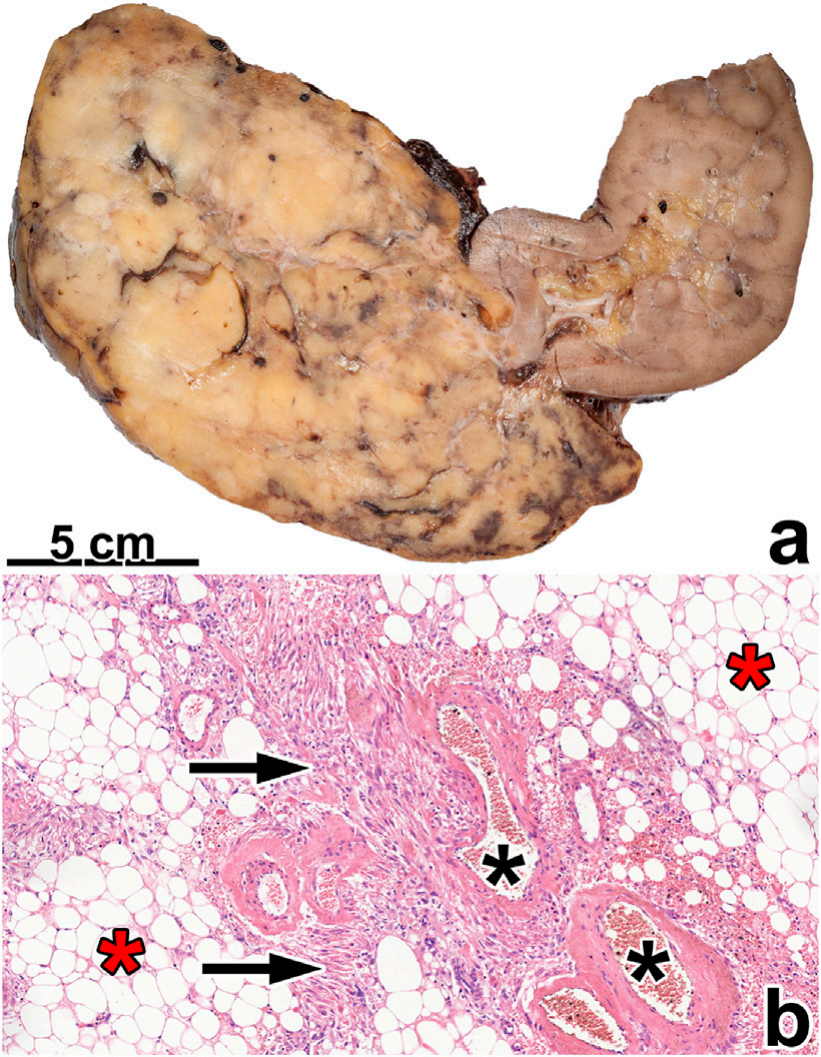
Classic angiomyolipoma. **(A)** The tumor is separated from the kidney parenchyma and has a fat tissue-like appearance. **(B)** Histologically, the tumor contains fat tissue (red asterisks), smooth muscle cells (black arrow), and thick blood vessels with hyaline walls (black asterisks). The image has a magnification factor of ×100.

Classic AML was the most common subtype (59.61%). Two tumors occurred in males (case #31 and #33). In case #35, the tumor developed in damaged kidney parenchyma (polycystic kidney), while, in case #33, the patient used finasteride to treat benign prostatic hyperplasia. All TSC-linked cases showed classic morphology. Figure 2 presents the morphological features of this subtype. Here, the median size of the tumors was 52 mm (range: 4–300 mm).

### Leiomyoma-like AML

**FIGURE 3:**
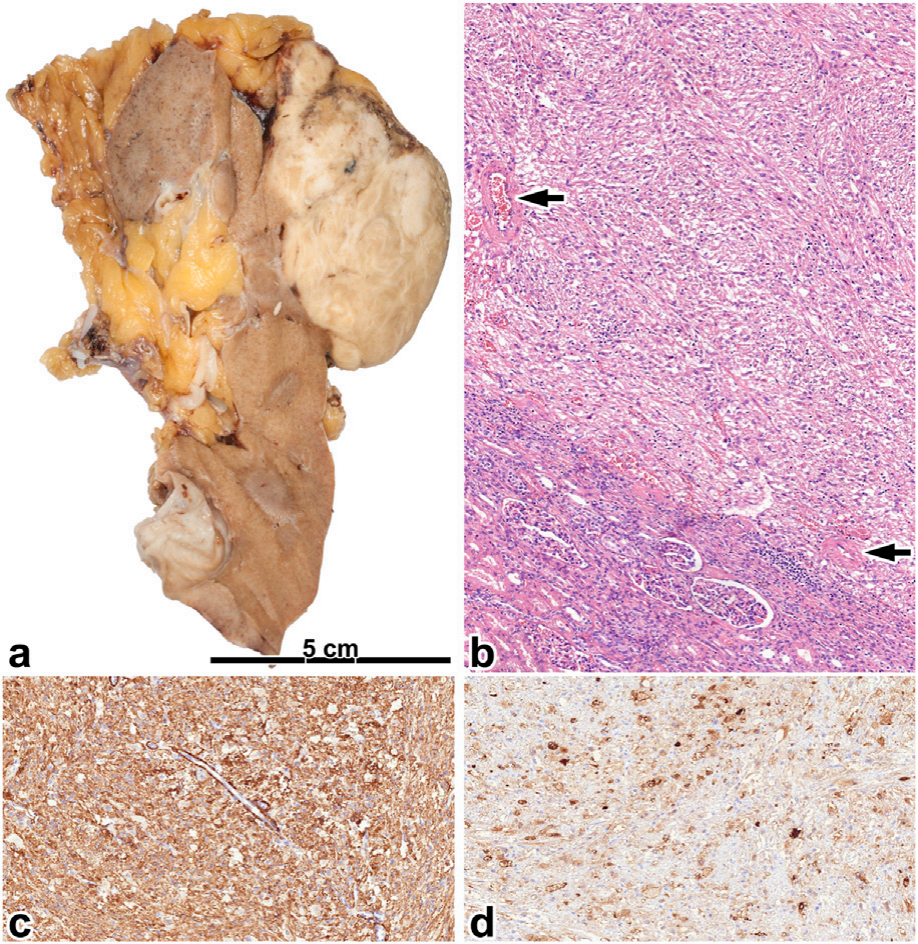
Leiomyoma-like angiomyolipoma. **(A)** Here, a greyish and whitish mass is present in the upper pole of the kidney, which is separated from the renal parenchyma and penetrates expansively to the adipose capsule. **(B)** The tumor is built up of spindle-shaped cells and blood vessels with thick walls (black arrow). The image has a magnification factor of ×100. **(C,D)** The SMA and MelanA immunostainings are diffusely positive. The two images have a magnification factor of ×200.

This morphology was seen in 25% of the AMLs investigated. These tumors contained only a small amount of fat tissue; therefore, macroscopically, they caused no impression of AML (shown in Figure 3).

### Lipoma-like AML

**FIGURE 4:**
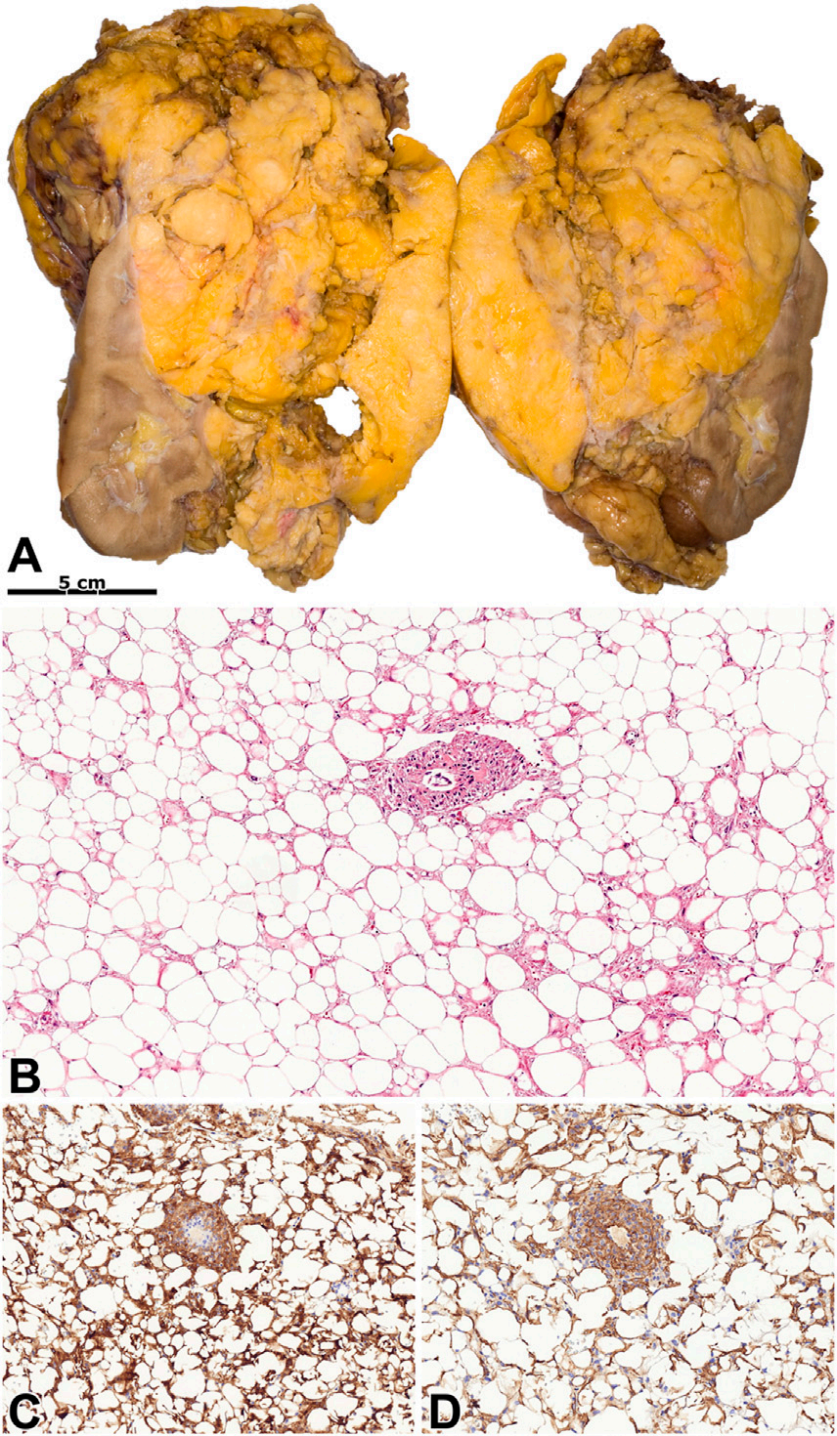
Lipoma-like angiomyolipoma. **(A)** An extensive mass with a fat-like appearance can be seen on the kidney’s cut surface. **(B)** The tumor histologically resembles lipoma, and blood vessels are occasionally seen. Besides, there are foci of smooth muscle cells among the adipocytes. The image has a magnification factor of ×100. **(C,D)** The tumor cells have diffuse co-expression (insert) of SMA and MelanA. The two images have a magnification factor of ×200.

We registered a lipoma-like appearance in five cases, and case #35 evolved in a transplanted kidney. Figure 4 summarizes the morphological characteristics of this subtype.

### Epithelioid AML

**FIGURE 5:**
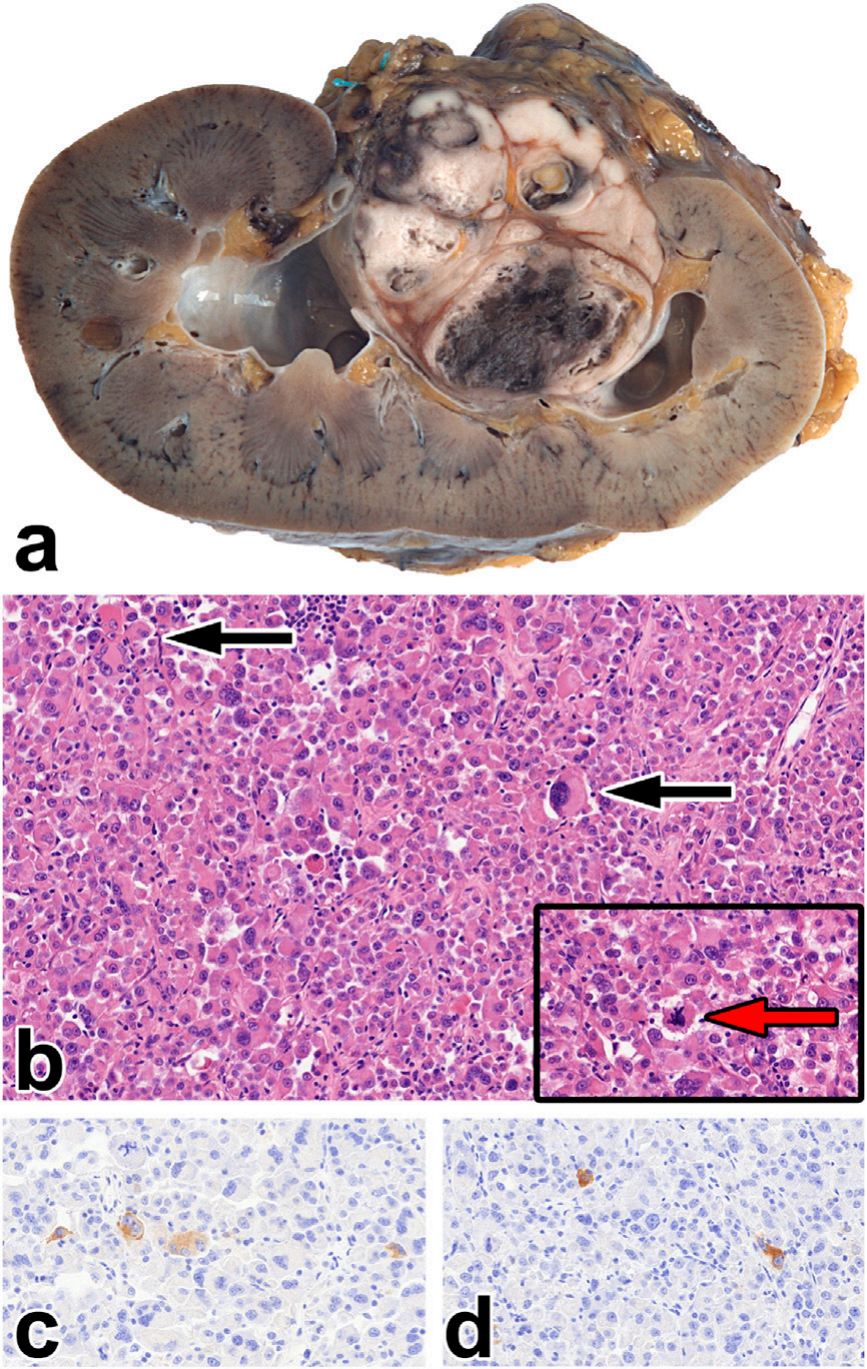
Epithelioid angiomyolipoma. **(A)** The gross picture shows a necrotic, hemorrhagic tumor located deeply in the renal sinus. **(B)** The tumor is made up of rhabdomyoblast-like cells. Besides, giant cells can be seen (black arrow), along with atypical mitosis (insert red arrow). The images have a magnification factor of ×200 and ×600, respectively. **(C,D)** MelanA and SMA co-expression is observed in some tumor cells. The two images have a magnification factor of ×400.

We diagnosed two tumors as eAML. This subtype required several immunohistochemical staining at the original histological diagnosis. Invasion, mitotic activity, and tumor cell necrosis were exclusively seen in these tumors (shown in Figure 5).

### AML with epithelial cysts

**FIGURE 6:**
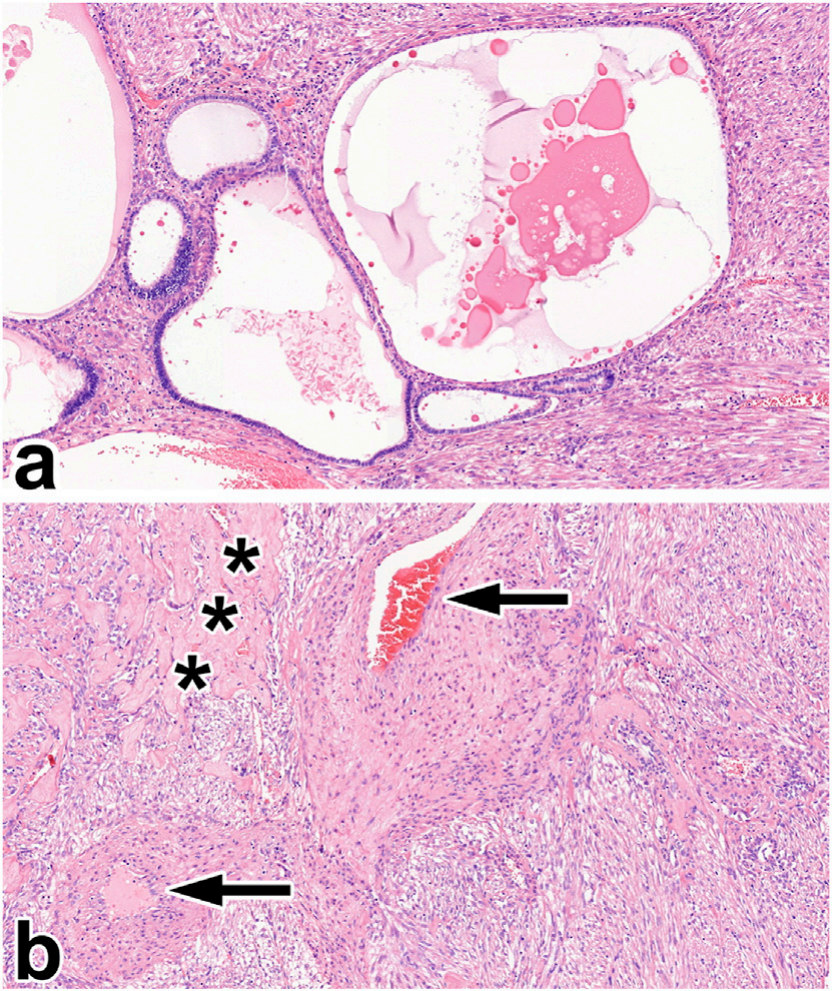
Angiomyolipoma with epithelial cysts. **(A)** The two-component tumor is built up of varying cysts in size and smooth muscle-rich stroma. **(B)** Apart from the smooth muscle cells, the stroma contains thick-walled blood vessels (black arrow) and sclerotic foci (black asterisks). All images have a magnification factor of ×100.

This tumor was discovered in a 46-years-old male. The relatively small lesion was accidentally noticed during a urological investigation. Histologically, the tumor shared some characteristics with metanephric stromal tumor, and the tumor cells were estrogen and progesterone receptor-positive. We have no data on any hormonal treatment. Figure 6 represents the histological features of AMLEC.

### Correlation with different renal neoplasms

We compared the gender, age and tumor size of AML patients with those, who were operated with oncocytoma (RO) and renal cell carcinoma (RCC). The AML is more common in females (AML vs. RO; *p* < .001; AML vs. RCC *p* < .001). Concerning the age among AML, onocytoma and RCC patients, the AML occurs 10 years and 7 years prior to the two aforementioned tumors, respectively (AML [mean age = 52.76] vs. RO [mean age = 62.41], *p* < .001; AML [mean age = 52.76] vs. RCC [mean age = 59.58], *p* < .001). There is no difference in terms of the tumor size among the three tumors (AML [mean size = 59.56 mm] vs. RO [mean size = 48.3 mm], *p* = .78; AML [mean size = 59.56 mm] vs. RCC [mean size = 62.61 mm], *p* = .571).

## Discussion

**FIGURE 7:**
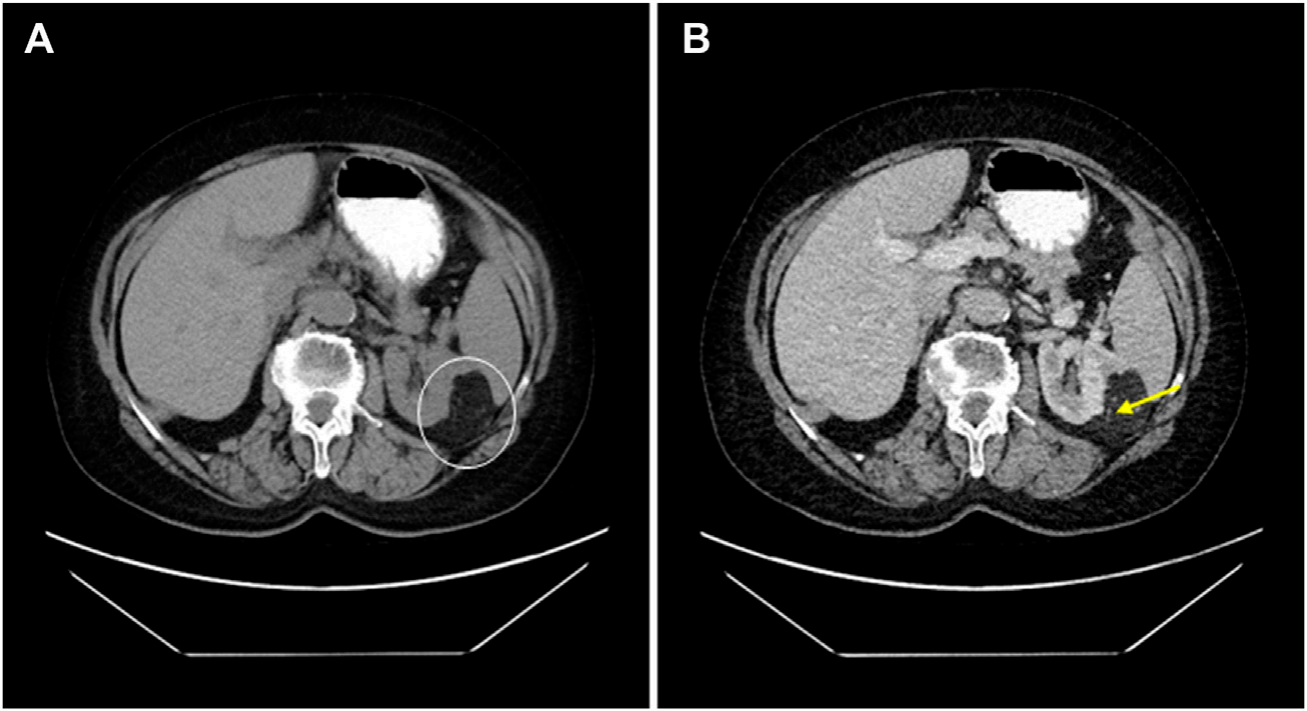
68-year-old female patient’s CT scan accidentally reveals an angiomyolipoma. **(A)** A native CT scan shows a lobulated 54 mm maximal axial diametric mass with a mean density of -79 HU (white circle). **(B)** In the same plane, the venous phasic cross-section shows that the difference does not change substantially; moreover, the thick-wall blood vessels become visible (yellow arrow).

Oncocytoma and AML are the most common benign tumors of the kidney [4]. The former originates from the collecting ducts, while the latter comes from the perivascular epithelioid cells; hence it belongs to the PEComa tumor group [4]. PEComas include lymphangioleiomyomatosis, lung clear cell (sugar) tumor, and abdominopelvic PEComa [20, 21]. These tumors have a myomelanocytic differentiation with an expression of smooth muscle and melanocytic markers [1,2,3,4]. Based on the literature data, approximately 1% of nephrectomies are carried out due to AML [14]. We had a slightly higher incidence rate (2.33%). The difference might have two causes. At first, the specimens studied are collected for a relatively long period of time (1978–2021), and secondly, during nearly 45 years, the availability and sensitivity of the imaging techniques evolved significantly. The AML can be sporadic or linked to TSC [4]. The former is more frequent in females (female-to-male ratio = approximately 4:1) [22, 23]. This difference might have hormonal causes since the tumor cells in AML can express hormone receptors; moreover, in pregnant, the underlying AMLs can start a rapid enlargement [6, 7, 24]. We also found female dominance in our material with a female-to-male ratio of 6.14:1. Some authors suggest the syndromic cases lack the gender difference [12], while others state the opposite [4]. In our case set, all AMLs linked to TSC involved females. AML can occur at any age, but sporadic cases are mostly diagnosed at the end of the fourth or in the beginning of the fifth decades [6, 8]. We had a similar observation: our sporadic cases’ mean age was 52.76, and on the other hand, TSC-linked AMLs developed 15 years earlier (mean age: 37.75). Also, we found that the sporadic AMLs appear approximately 10 years earlier than RCC; therefore, in unsure renal tumor cases, for female patients in this age group, a renal biopsy can be advised to achieve the best patient care. For childhood AML cases, genetic consultation and studies are required [8]. AMLs usually have no symptoms, and they are incidental findings of examinations for other underlying disorders (i.e., hypertension, staging of malignant tumor) [4, 25]. According to the literature data, tumors larger than 40 mm induce symptoms in 80% of the cases. The common symptoms are groin pain, hematuria, and newly recognized high blood pressure [26]. Hemorrhagic complications are detected more frequently in tumors larger than 40 mm [12, 25, 27]. Limited anamnestic data were available in our study. A hemorrhagic complication was reported in eight patients, one of whom developed a life-threatening hemorrhagic shock. The average size of such tumors in our material was 110 mm. Most AMLs are radiologically safe to diagnose. Like the pathological classification, imaging diagnostics classify AML into several groups, including fat tissue-rich AML, fat tissue-poor AML, and fat tissue-invisible [28]. The fat tissue-rich AML is the largest group, corresponding to the classical type known from the pathological classification. By B-mode ultrasound, fat tissue-rich AML is described as a typical, homogeneous, hyperechoic lesion with no signs of necrosis or calcification [28]. The non-enhanced CT scan also gives a typical picture (shown in Figure 7) because one can measure fat density, i.e., values below -10 HU [29]. The MRI examination may help to distinguish between AML and RCC, as the loss of signal intensity between in-phase and out-of-phase sequences indicates the presence of microscopic fat tissue [30]. The tumor is usually separated from the kidney parenchyma, renal sinus, and adipose capsule but may appear in the renal vein or the regional lymph nodes [26, 31, 32]. The latter refers to a multicentric origin rather than metastasis [33]. The composition of the tumor influences the macroscopic appearance. The classic and lipoma-like AMLs have an adipose tissue-like cut surface, whereas leiomyoma-like AML and AMLEC mostly form a greyish-whitish mass [34]. The tumor is usually unilateral and unifocal, but 1/3 of the cases are multifocal, and 15% of the AMLs are bilateral [35]. Seven of our cases had multiple foci, and one patient with TSC had a bilateral tumor. In classic AML, the tumor consists of fat tissue, smooth muscle tissue, and thick-walled, irregular blood vessels in nearly similar proportions [36]. The adipose component corresponds to mature fat tissue, but sometimes vacuolated cells resembling lipoblasts can be detected. The smooth muscle cells typically form irregular fascicles and circular growth from the vessel wall [37]. The cytological atypia is usually mild, but sometimes, bizarre and multinucleated cells are present. The vascular component resembles thick-walled artery-like vessels with a thin elastic layer [4, 38]. The pathological diagnosis is straightforward in classic morphology, and immunostainings are unnecessary. Leiomyoma-like AMLs are often located below the fibrous capsule [39]. Because of the low-degree fat tissue component, the possibility of leiomyoma or schwannoma may arise; however, these, like other benign soft tissue tumors, are rare in the kidney and lack a myomelanocytic immunophenotype [40, 41]. In contrast to the former subtype, in the lipoma-like AML, the smooth muscle component may have a small amount, and the tumor is composed almost exclusively of fat tissue [42]. Such tumors are often associated with the adipose capsule and should be discriminated from lipoma or atypical lipomatous tumor [43]. These tumors are not characterized by myomelanocytic immunophenotype; furthermore, overexpression of MDM2 and CDK4 are seen in the latter [44]. The AMLEC is composed of stromal and epithelial components. The former is neoplastic, typically with irregular, smooth muscle bundles. The epithelial component forms cysts of varying size, but these most likely correspond to entrapped and dilated nephron segments [45, 46]. Currently, the term of angiomyolipoma with epithelial cysts is used instead of cystic AML [47]. This subtype is characterized by hormone receptor positivity, and accordingly, the tumor should be distinguished from the mixed epithelial and stromal tumor of the kidney (MESTK) [45]. The stroma of the two entities differs because, in MESTK, it mimics the ovarian stroma. Also, MESTK has no co-expression of melanocytic and smooth muscle markers [48]. From a clinical point of view, the identification of eAML is crucial, because in contrast to the subtypes discussed so far, this variant may recur in some cases and give metastases [49]. The incidence of malignant behavior is quite different in the literature, but the likelihood is approximately 5% based on two large-number studies [37, 49]. Some epithelioid component is present in all AMLs, but if its proportion is above 80%, the tumor must be diagnosed as eAML [49, 50]. The pure eAML is rare and can cause severe diagnostic difficulties as it should be distinguished mainly from rhabdoid RCC, translocation RCC, primary renal pleomorphic sarcoma, and metastasis; therefore, a large amount of immunohistochemistry is usually performed [51, 52]. Of note, the myomelanocytic phenotype can also be observed, but it is often present focally [49]. Genetically, all types of AML are characterized by the inactivation of the *TSC1* or *TSC2*, which impairs the regulation of the mTOR signaling pathway and leads to increased cell proliferation [53]. A *TP53* mutation has also been noted in eAML, possibly contributing to the malignant clinical course [54]. Genetic testing is only required if TSC is suspected. Immunomorphologically, AML is usually diffusely positive with SMA, and other muscle markers expression is seen in 50% of cases [49]. Expression of a melanocytic marker is also always observed, most commonly MelanA or HMB45. In a comparative study of 20 cases, all AMLs were diagnosed using these two markers, and other melanocytic markers (tyrosinase, CD117, NK1-C3, etc.) have little diagnostic benefit [55]. Focal SMA staining was present in our material only in eAML, and 75.6% of the study cases expressed both melanocytic markers, of which MelanA immunostaining was generally more extensive and more often positive. Immunohistochemistry of hamartin and tuberin has no diagnostic value [56]. The treatment is influenced by tumor size, bilaterality, and the possibility of malignancy. Asymptomatic tumors below 40 mm should be monitored by annual CT or MRI [57]. A closer observation is recommended in the range of 40–80 mm; usually, surgical treatment is performed in half of the patients due to complications [57]. Surgical removal should be advised in symptomatic cases, preferably using nephron-sparing techniques [58]. The tumors larger than 80 mm have a high risk of hemorrhagic complications and should be treated surgically or with radiologic intervention, such as selective arterial embolization or radiofrequency ablation [59]. The complications and stress are less severe in intervention, but these patients should be followed because tumors may recur [60]. To maintain renal function, a nephron-sparing resection is also preferred in bilateral cases [61]. In doubtful cases, a biopsy is required as a first step, and depending on the diagnosis, observation or surgery should be carried out [57, 62]. Systemic treatment is used for metastatic eAML, which may be chemotherapy, mTOR inhibitors, or immunotherapy [63, 64].

## Conclusion

AML is rare in nephrectomy specimens, and it is either sporadic or associated with TSC. It typically develops in women around 50 years, and the average size of surgically treated cases is about 50–60 mm. Histologically, the tumor may be classic, leiomyoma-like, lipoma-like, epithelioid, or cystic, in order of frequency. The eAML may be malignant, so such tumors should be treated like RCC and closely monitored. The pathological diagnosis is usually problem-free, and the light-microscopic findings may be supplemented by MelanA, HMB45, and SMA immunostaining.

## Data Availability

The raw data supporting the conclusion of this article will be made available by the authors, without undue reservation.
